# *Arlenea delicata* gen. et sp. nov., a new ephedroid plant from the Early Cretaceous Crato Formation, Araripe Basin, Northeast Brazil

**DOI:** 10.1016/j.pld.2023.06.008

**Published:** 2023-06-30

**Authors:** Alita Maria Neves Ribeiro, Yong Yang, Antônio Álamo Feitosa Saraiva, Renan Alfredo Machado Bantim, João Tavares Calixto Junior, Flaviana Jorge de Lima

**Affiliations:** a Programa de Pós-Graduação em Diversidade Biológica e Recursos Naturais, Universidade Regional do Cariri, Rua Carolino Sucupira, Pimenta, 63105-160, Crato, Ceará, Brazil; b Co-Innovation Center for Sustainable Forestry in Southern China, College of Biology and the Environment, 159 Longpan Road, Nanjing Forestry University, Nanjing, 210037, China; c Laboratório de Paleontologia, Universidade Regional do Cariri, Rua Carolino Sucupira, Pimenta, 63105-160, Crato, Ceará, Brazil; d Laboratório de Estudos da Flora Regional, Universidade Regional do Cariri, Rua Carolino Sucupira, Pimenta, 63105-160, Crato, Ceará, Brazil; e Gondwanan Plants Lab, Universidade Federal de Pernambuco, Centro Acadêmico de Vitória, Rua Do Alto Reservatório S/n, Bela Vista, 55608-680, Vitória de Santo Antão, Pernambuco, Brazil; f Programa de Pós-Graduação em Geociências (PPGEOC), Universidade Federal de Pernambuco (UFPE), Rua Av. da Arquitetura, S/nº CEP - 50740-550, Cidade Universitária, Recife, Pernambuco, Brazil

**Keywords:** Early Cretaceous, Gnetophytes, *Arlenea delicata*, Ephedrales, Crato Formation

## Abstract

Ephedroid macrofossils have been widely documented in Cretaceous deposits, including numerous from the Lower Cretaceous Yixian Formation of NE China. However, few ephedroid macrofossils have been reported from South America. Herein, we describe a new plant of the family Ephedraceae, *Arlenea delicata* gen. et sp. nov., from the Lower Cretaceous Crato Formation of the Araripe Basin, Northeast Brazil, based on the vegetative and reproductive structures. It has the typical morphological characteristics of ephedroid plants, including fertile reproductive branches, opposite phyllotaxy, terminal female cones, a sympodial branching system, longitudinally striated internodes, and swollen nodes. Our new finding is unusual in having inner chlamydosperms subtended by two pairs of bracts, reproductive units connected to branches through swollen receptacles and a smooth seed surface. This new ephedroid taxon from the Crato Formation increases our understanding of plant diversity of this group during the Early Cretaceous. Furthermore, the general morphology (fleshy bracts and enlarged receptacles) of this new fossil discovery indicates that seeds of this plant may have been dispersed by animals such as pterosaurs (mainly the Tapejaridae) and birds (Enantiornithes and Ornituromorpha). If true, this would explain the cosmopolitan distribution of Ephedraceae in the Lower Cretaceous.

## Highlights

• *Arlenea delicata* it's a Ephedraceae with bearing female reproductive units, with vegetative and reproductive characters.
• The macro and micromorphology of *A. delicata* suggest that it developed in places with a water deficit.
• The shape of the enlarged receptacle, as well as the bract type, indicates that *A. delicata* seeds may have been dispersed by animals.

## 1 Introduction

Modern gnetophytes consist of three monogeneric families: the Ephedraceae, Gnetaceae and Welwitschiaceae. The family Ephedraceae consists of a single genus (*Ephedra* L.) which contains 70 shrub/subshrub species with striated, photosynthetic branches, reduced leaves in the form of scales, as well as female and male strobiles (Price, 1996; Yang et al., 2005, 2017, 2022; Santos and Chow, 2014a). Furthermore, Ephedraceae have branches that are often ramified profusely and arranged oppositely or ternately whorled at the nodes (Pearson, 1929; Mussayev, 1978). The branches are cylindrical and articulated, while the internodes have numerous longitudinal striations (Yang et al., 2005). These plants are dioecious or rarely monoecious, have ovoid to ellipsoidal cones that may be terminal or axillary, and bear one to three seeds. The female cones bear bracts arranged in 2–9 pairs or whorls of three, of which only the upper ones are fertile (Stapf, 1890; Cutler, 1939; Stevenson, 1993). The bracts are membranous, leathery, or fleshy at maturity. The seed surface is smooth or carved in distinct species (Yang et al., 2005; Yang, 2011). Plants of the family are widely distributed in temperate arid areas of Eurasia, northern Africa, southwestern North America, and western South America (Price, 1996; Yang et al., 2005; Yang, 2007).

The fossil record is replete with gnetophytes, which reached peak diversity in the Early Cretaceous before abruptly declining to near-extinction by the end of the Cretaceous (Crane and Lidgard, 1989; Crane, 1996; Osborn, 2000; Rydin et al., 2006). Many fossils related to *Ephedra* sp. from the Early Cretaceous have been reported from Southern Europe, Northeast China, Mongolia, as well as North and South America (e.g., Guo and Wu, 2000; Yang et al., 2005; Rydin et al., 2006; Wang and Zheng, 2010). Among these records, we highlight the seeds with *in situ* pollen from Portugal (Figueira da Foz Formation, Early Cretaceous) and North America (Patuxent Formation, Early Cretaceous) (Rydin et al., 2006), reproductive branches in Argentina (Anfiteatro de Ticó Formation, Early Aptian) (Cladera et al., 2007), and China (Yixian Formation, Lower and Upper Cretaceous) (Yang et al., 2005; Rydin et al., 2006; Liu et al., 2008; Rydin and Friis, 2010; Wang and Zheng, 2010; Yang, 2010; and many other papers), and a branch probably containing male strobili in Brazil (Crato Formation, Lower Cretaceous) (Kerkhoff and Dutra, 2007). Early ephedroid plants show high species diversity in the Yixian Formation of northeastern China, but have been documented less frequently in South America.

Gnetophyte fossils found in the Crato Formation, Lower Cretaceous of the Araripe Basin, have constituted an important contribution to our understanding of Gnetales, bringing more information about this group in relation to the geological past, helping to understand the current diversity of this group of plants. This paper presents of a new species of fossil plant related to Ephedraceae with information on vegetative and reproductive characters, expanding our knowledge about the diversity and biogeography of this family in the global fossil record.

## 2 Material and methods

### 2.1 Geological setting

**Fig. 1:**
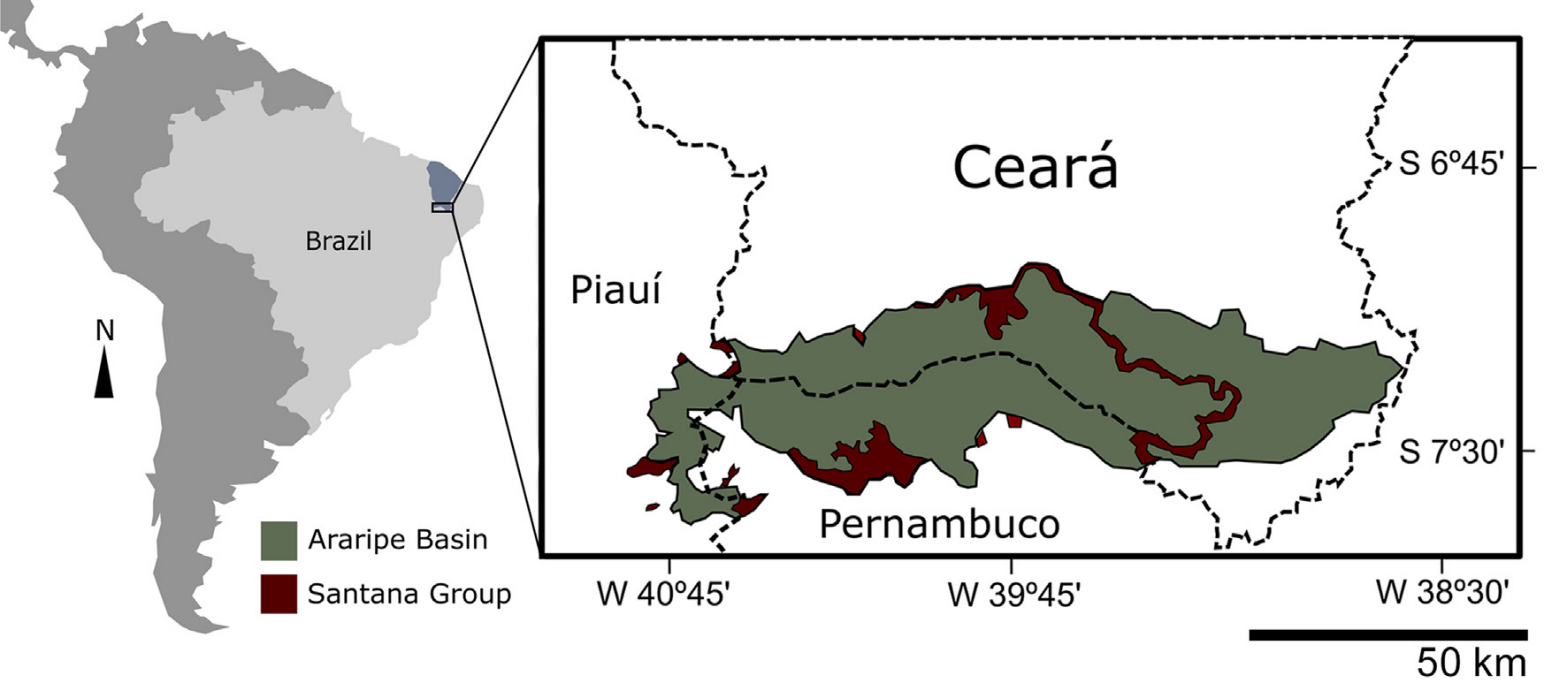
Location of the Araripe Basin in South America, bordering the states of Ceará, Piauí and Pernambuco in northeastern Brazil.

The Araripe Basin is located at the “Borborema” Structural Province (Assine et al., 2014), which covers practically the entire Northeast of Brazil, with rocks from the Precambrian basement (Valença et al., 2003). This sedimentary basin is located between 38°30’ to 40°50’W and 7°05’ and 7°50’S, occupying part of the states of Piauí, Ceará and Pernambuco (Assine, 2007) (Fig. 1). The main lithostratigraphic unit of the Araripe Basin is the Santana Group (Neumann and Cabrera, 1999), which represents the Barbalha, Crato, Ipubi and Romualdo formations, with Crato and Romualdo standing out for their quality, quantity and preservation of the fossiliferous content found in them, which gives them the title of *fossil-lagerstätten* (Maisey, 1991; Martill, 2007).

The Crato Formation outcrops on the northeast flank of the Araripe Basin, constituting an important geological and paleontological site in Brazil (Viana and Neumann, 2002). This formation consists of laminated micritic limestones, with a color ranging from gray to yellow with halite pseudomorphs (Neumann and Cabrera, 1999). Several levels of the Crato Formation contain fossils, including those of arthropods, fish, amphibians, chelonians, crocodylomorphs, pterosaurs, squamata, ostracods, conchostraceans, birds, as well as pollen, spores, and plants (Saraiva et al., 2021).

### 2.2 Crato Formation gnetophytes

Because of their exceptional preservation, the fossil plants from the Crato Formation, Lower Cretaceous of the Araripe Basin, represent one of the few floras of northern Gondwana that has been studied continuously for many years and therefore provides a relatively detailed overview of the composition and diversity of flora in this paleoequatorial area to which it was inserted (Friis et al., 2011).

Gnetophytes from the Crato Formation are very common and diverse. The first records of gnetophyte macrofossils that were provisionally assigned to Ephedraceae and Welwitschiaceae for the Crato Formation were recognized by Pons et al. (1992) and Bernardes-de-Oliveira et al. (1999, 2000). Kerkhoff and Dutra (2007) identified an ephedroid form named *Ephedra paleoamericana* Kerkhoff and Dutra, a branch probably containing male strobili. Ricardi-Branco et al. (2013) described *Itajuba yansanae* Ricardi-Branco et al., which has a branch system containing terminal female cones (Ricardi-Branco et al., 2013). *Cearania heterophylla* Kunzmann et al. is considered to be a possible gymnosperm with gnetalean affinities and terminal strobilus surrounded by numerous bracts (Kunzmann et al., 2009). *Friedsellowia gracilifolia* Löwe et al. (2013) exhibits a gnetalean habit and terminal reproductive organs consisting of cones (Löwe et al., 2013). The Welwitschiaceae family is represented by some species, such as *Cratonia cotyledon* Rydin et al. which consist of two large oval cotyledons (Rydin et al., 2003); *Priscowelwitschia austroamericana* Dilcher et al. (2005) a welwitschioid vegetative and reproductive structures; isolated leaves of *Welwitschiophyllum brasiliense* Dilcher et al. (2005); and male strobiles of *Welwitschiostrobus murili* Dilcher et al. (2005).

### 2.3 Study material

The fossils analyzed here consist of impressions and three-dimensional preservation of fragmented fertile reproductive branches from the Crato Formation, Araripe Basin, Northeastern Brazil, deposited in the collection of the Museu de Paleontologia Plácido Cidade Nuvens (MPPCN), in Santana do Cariri municipality, Ceará, Brazil. We analyzed four specimens representing the female plant, with vegetative and reproductive structures: MPSC PL 3863, MPSC PL 3862, MPSC PL 5250 p/cp (part and counterpart) and MPSC PL 635.

The specimens were analyzed at the Laboratory of Paleontology of the Universidade Regional do Cariri (LPU), in Crato, Ceará, where the mechanical preparation was carried out, which consisted of removing the matrix rock until the appearance of the specimen with the greatest number of observable characteristics, without compromising its integrity. The specimens were photographed with a Canon EOS 60D Camera - DS126281 and a Fluorescence Stereo Zoom Microscope, Axio Zoom. v.16, to photograph details of the female reproductive units of the specimens.

The samples were analyzed with a desktop Scanning Electron Microscope (Phenom XL, Thermo Fisher Scientific) of the Laboratório de Micropaleontologia Aplicada - Universidade Federal de Pernambuco (LMA-UFPE). For this purpose, we removed and mounted reproductive structures and branches from each specimen.

Here, we classified the ovulated units of the female cones as “female reproductive units” (FRUs), following the work of Yang (2001). For systematic classification, we followed the model by Yang et al. (2022), which summarizes new advances in the phylogeny of gymnosperms and proposes an updated classification of extant gymnosperms in which Ephedraceae are part of the Ephedrales order.

## 3 Results

### 3.1 Systematic palaeontology

**Order** Ephedrales Dumortier 1829

**Family** Ephedraceae Dumortier 1829

**Fossil Genus**. *Arlenea* Ribeiro, Yang, Saraiva, Bantim, Calixto Junior et Lima gen. nov.

**Etymology**. Genus (new) *Arlenea* in honor of a professor and botanist at the Universidade Regional do Cariri, Maria Arlene Pessoa da Silva PhD, for his tireless work in the research and protection of the extant flora of Chapada do Araripe.

Plant fossil names registry number. urn:lsid:ipni.org:names:77322502-1

**Type species**. *Arlenea delicata* Ribeiro, Yang, Saraiva, Bantim, Calixto Junior et Lima sp. nov.

**Generic diagnosis**. Reproductive branches fertile, aphyllous, with terminal female cones, sympodial branching system, internodes longitudinally striated, with swollen nodes, from which 2–3 ramifications depart. Opposite branches of phyllotaxis. Solitary and terminal reproductive structures with oblong-oval to ovoid shape and apparently obtuse to cuspidate apex, show swollen receptacle, two pairs of bracts surrounding two ovals inner chlamydosperms, possibly longitudinally striated. Elongated ovoid to oblong-oval seed shape.

**Fig. 2:**
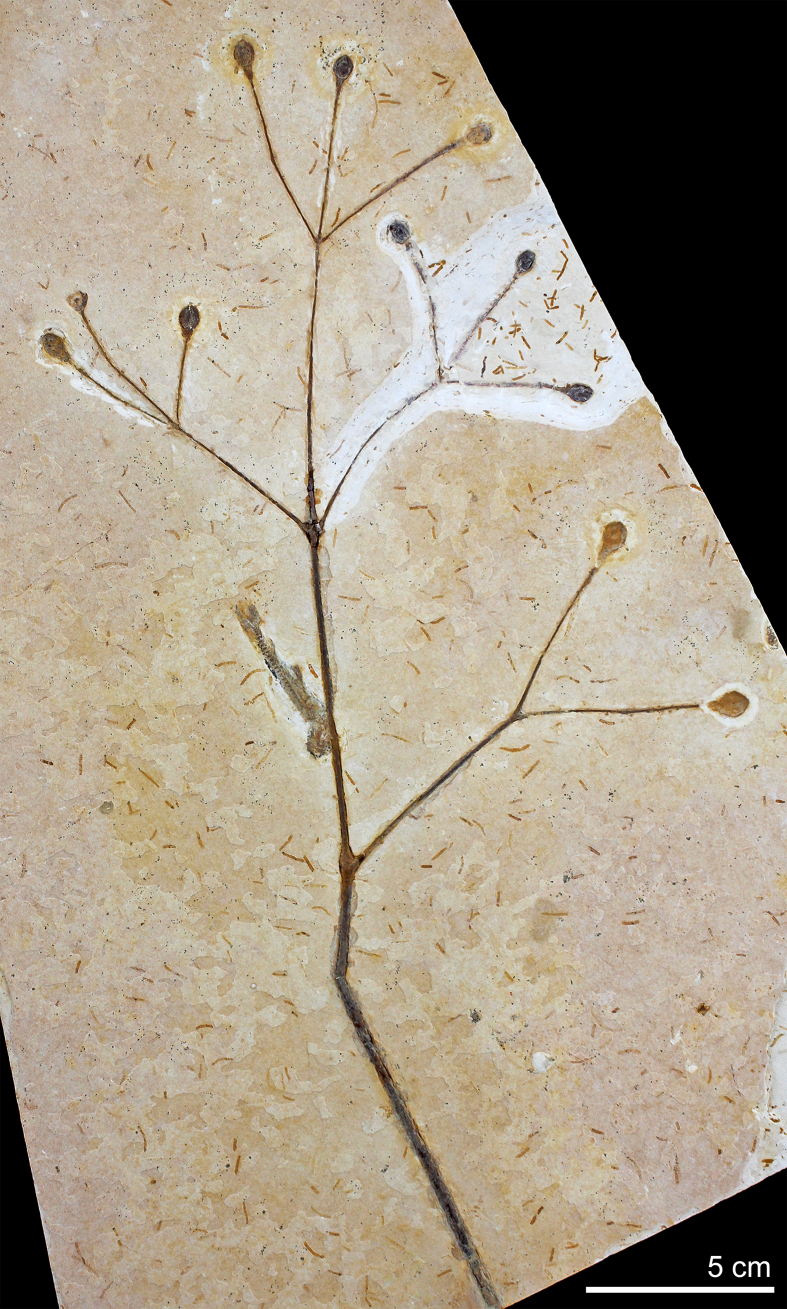
MPSC PL 3863, the holotype of *Arlenea delicata* gen. et sp. nov. showing the general morphology of the species. Scale bar = 5 cm.

**Fig. 3:**
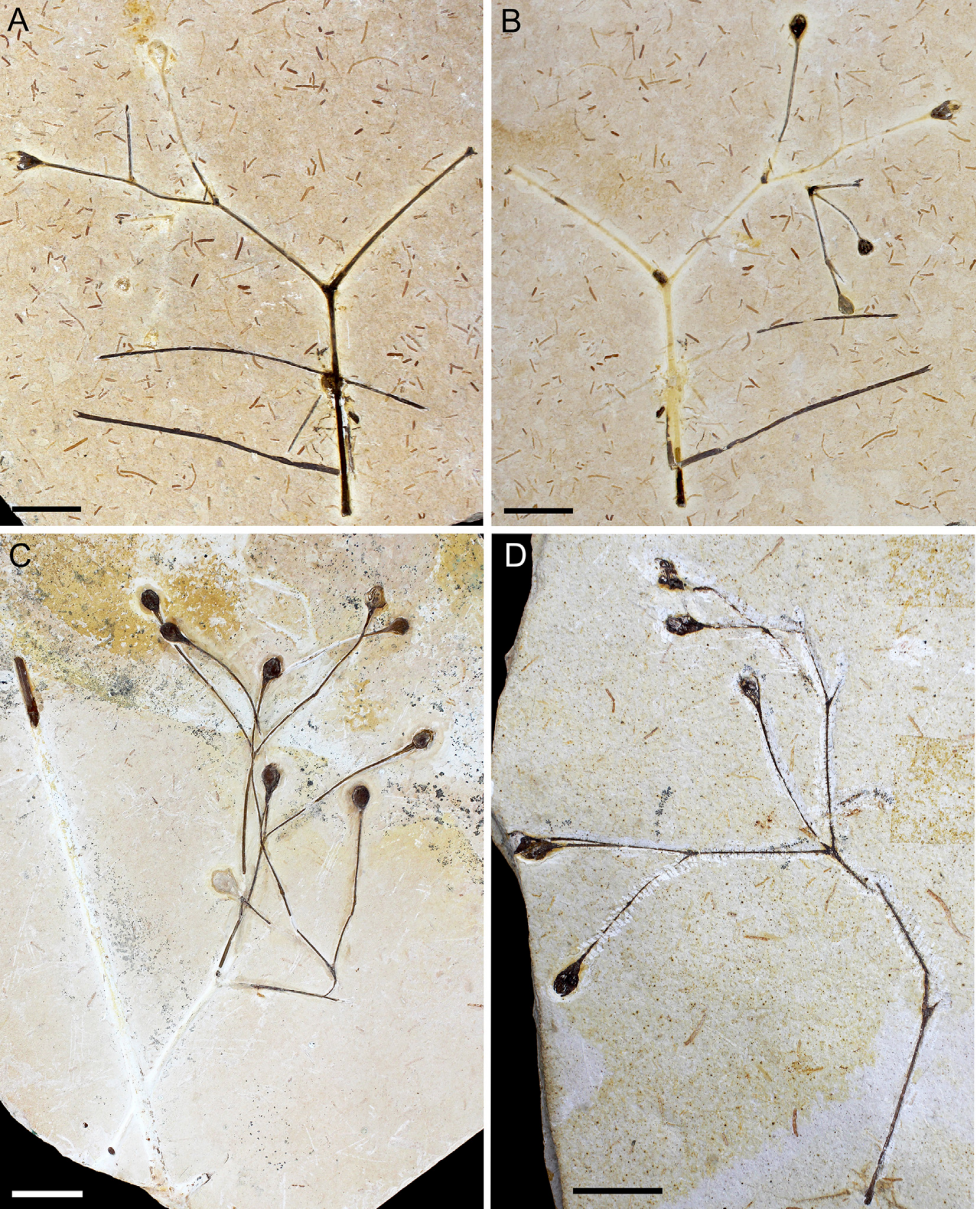
A, B: Paratype (MPSC PL 5250 p/cp) showing the general morphology, with terminal FRUs. C: Paratype (MPSC PL 3862). D: Paratype (MPSC PL 635). Scale bar = 2 cm.

**Fig. 4:**
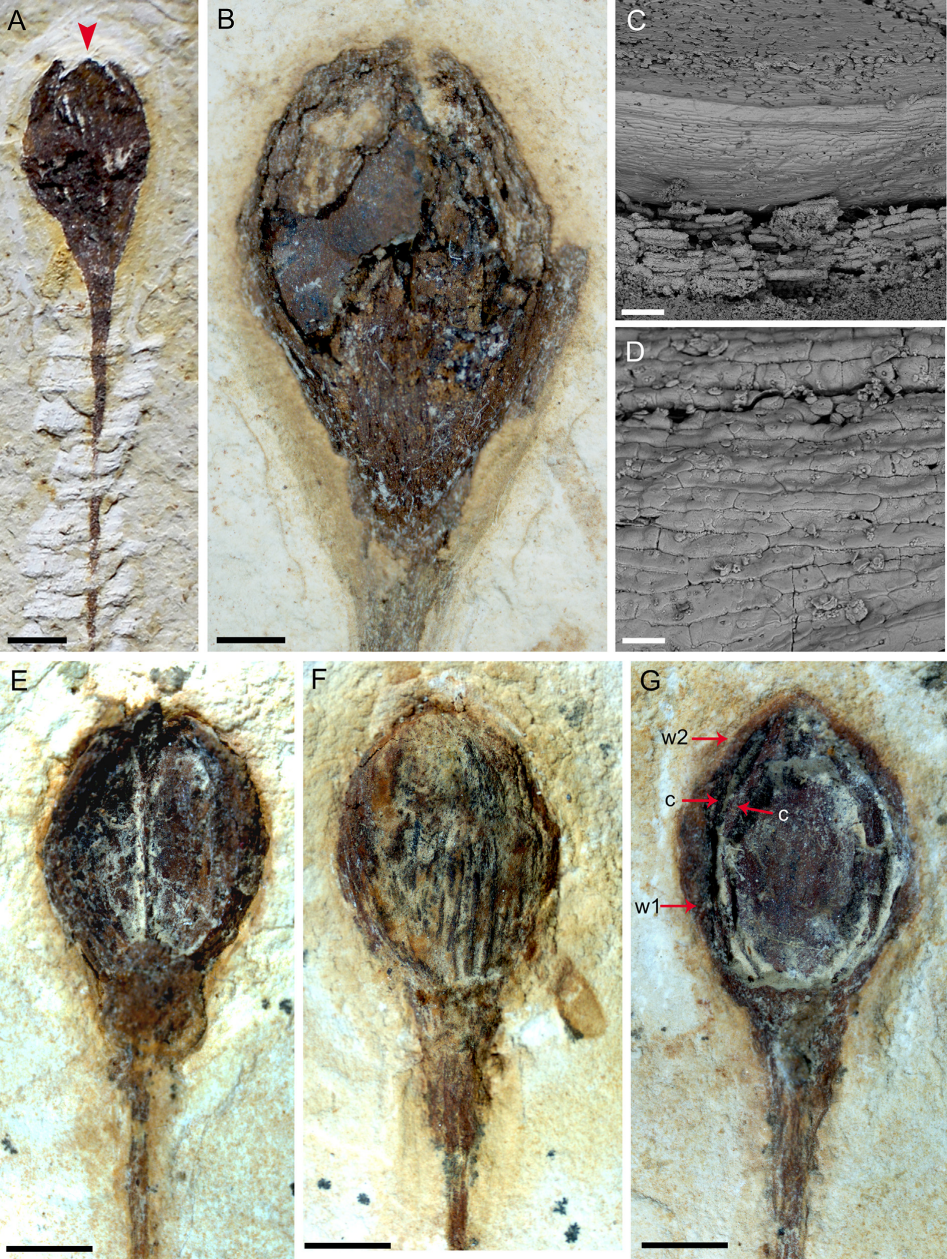
A: FRU of MPSC PL 635 showing cuspidate apex (indicated by red arrow). Scale bar = 2 mm; B: FRU of MPSC PL 5250 p/cp showing fragmented seed with a rounded base and oval apex. Scale bar = 1 mm. C: SEM image of MPSC PL 3863 FRU, with convex upper face. Scale bar = 100 μm; D: SEM image of MPSC PL 3863 reveals the convex periclinal walls on the outer surface of the seed. Scale bar = 40 μm; E–F: FRU examples of *Arlenea delicata* gen. et sp. nov. with different preservation sides, lateral and dorsal respectively. Scale bar = 2 mm; G: MPSC PL 3863 FRU with two pairs of proximal and distal bracts. The distal pair is long and fused, completely enveloping two internal chlamydosperms, oval, and swollen receptacle. Scale bar = 2 mm (Abbreviations: w1, proximal whorl of bracts; w2, distal whorl of bracts; c, chlamydosperms).

**Fig. 5:**
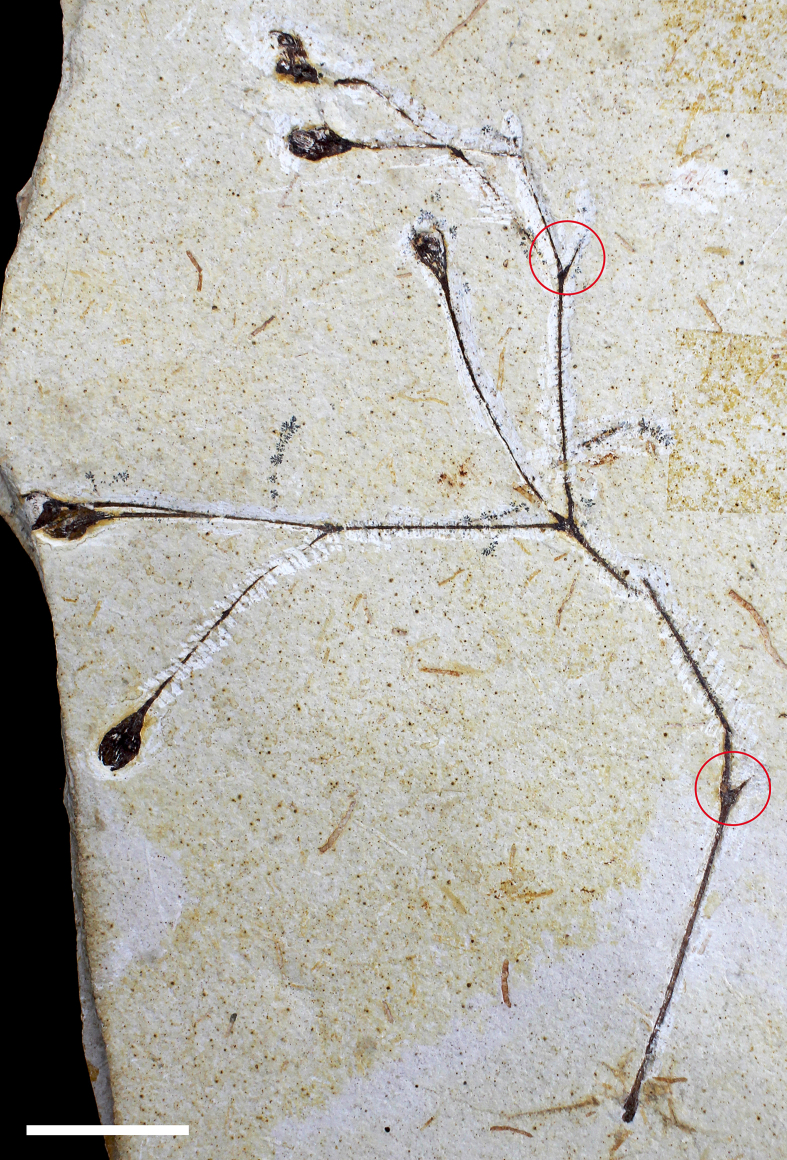
MPSC PL 3862 specimen with the side buds, highlighted in red circles. Scale bar = 2 cm.

*A. delicata* Ribeiro et al. sp. nov (Fig. 2, Fig. 3, Fig. 4, Fig. 5).

**Etymology**. The epithet “*delicata*” reflects the delicate terminal branches that contain terminal reproductive structures.

**Diagnosis**. As the same for the genus.

**Holotype**. MPSC PL 3863 (Fig. 2).

**Paratypes** designated here. MPSC PL 3862, MPSC PL 5250 p/cp, MPSC PL 635 (Fig. 3).

**Repository**. Palaeobotanical collection of the Museu de Paleontologia Plácido Cidade Nuvens (MPPCN), in Santana do Cariri municipality, Ceará, Brazil.

**Type locality**. Araripe Basin, Northeast Brazil.

**Type horizon**. Crato Formation, Lower Cretaceous.

**Description**: Incomplete reproductive branches, longitudinally striated and of opposite phyllotaxis. 2–3 branches depart from the nodes, bifurcate and trifurcate and are visibly swollen, articulated, with fine longitudinal striations (Fig. 2). The internodes are short to long, proportional to the size of the plant. The reproductive units found in *Arlenea* *delicata* are solely female, possibly bearing seeds, thus, representing a female dioecious plant, with FRUs terminal to branches, solitary, ovoid uni-ovulated and unilocular with two pairs of bracts in an apical pattern of dicasial branching, with seed inserted in the axil of the upper bract. The seed probably has a rounded base and oval to cuspidate apex in some FRUs, smooth surface, without transverse and undulating protuberances, with fine longitudinal grooves (Fig. 4c and d), upper surface convex, periclinal walls convex on the face of the outer surface of the seed (Fig. 4c and d). The FRUs are preserved in lateral and dorsal views (Fig. 4e and f). The micropylar tube, the tubular structure of the seed in live gnetophytes resulting from the elongation of the integument of the ovules in the apical portion, seems to be included in our species, seen by the elongated apex observed in the FRUs where the micropylar tube is possibly inserted (Fig. 4g).

The two pairs of bracts, proximal and distal, are completely enveloped by two internal chlamydosperms (Fig. 4g), ovoid and longitudinally striated. The bracts have a long-acuminate termination and are denser in the median portion than at the base, where they are inserted into a less slender receptacle, but continuous and dilated in relation to the stem axis (4.1 mm × 2.2 mm), with longitudinal grooves.

**Fig. 6:**
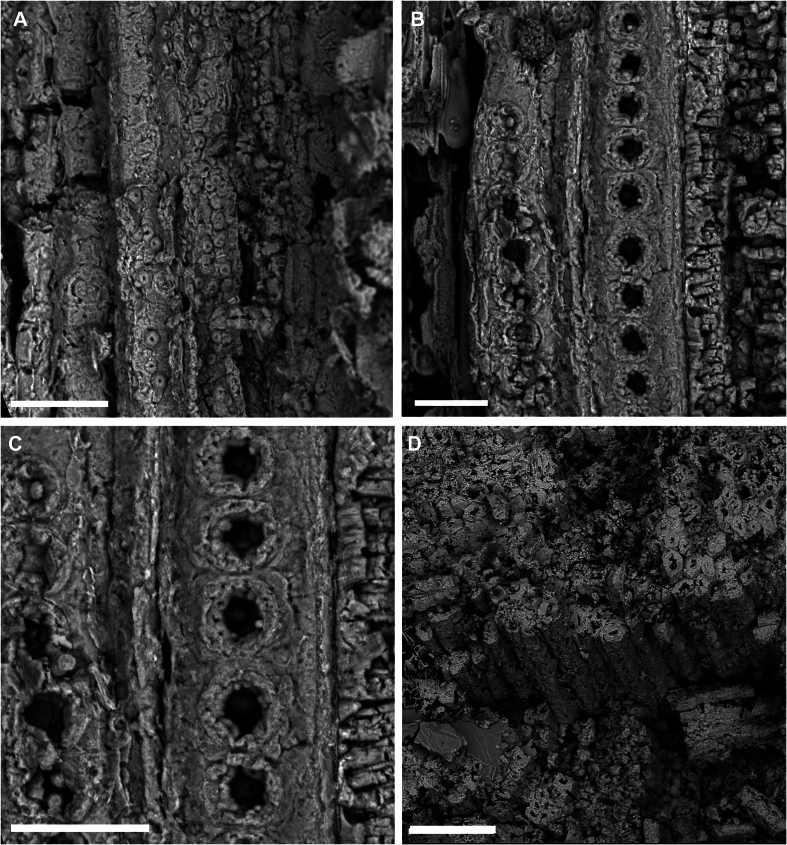
A: Longitudinally areolated pits distributed within tracheids. Scale bar = 50 μm; B: Vessel elements with perforation plates and areolated pits seen inside. Scale bar = 25 μm; C: Vessel elements with perforation plates and areolated pits seen inside (detailed view). Scale bar = 30 μm; D: Abundant tracheids seen in vertical section of the basal part of the branch of *Arlenea delicata* holotype. Scale bar = 100 μm.

Starting from the node of the lower portion of the MPSC PL 635 paratype, there is probably a lateral bud, as a continuation of the shoot projection in possible development for the formation of a new branch, as is also observed in one of the nodes of the upper portion of the specimen (Fig. 5). The branches have abundant tracheids with numerous longitudinally distributed areolated pits and vessel elements with perforation plates and internal areolated pits (Fig. 6a–d).

## 4 Discussion

**Fig. 7:**
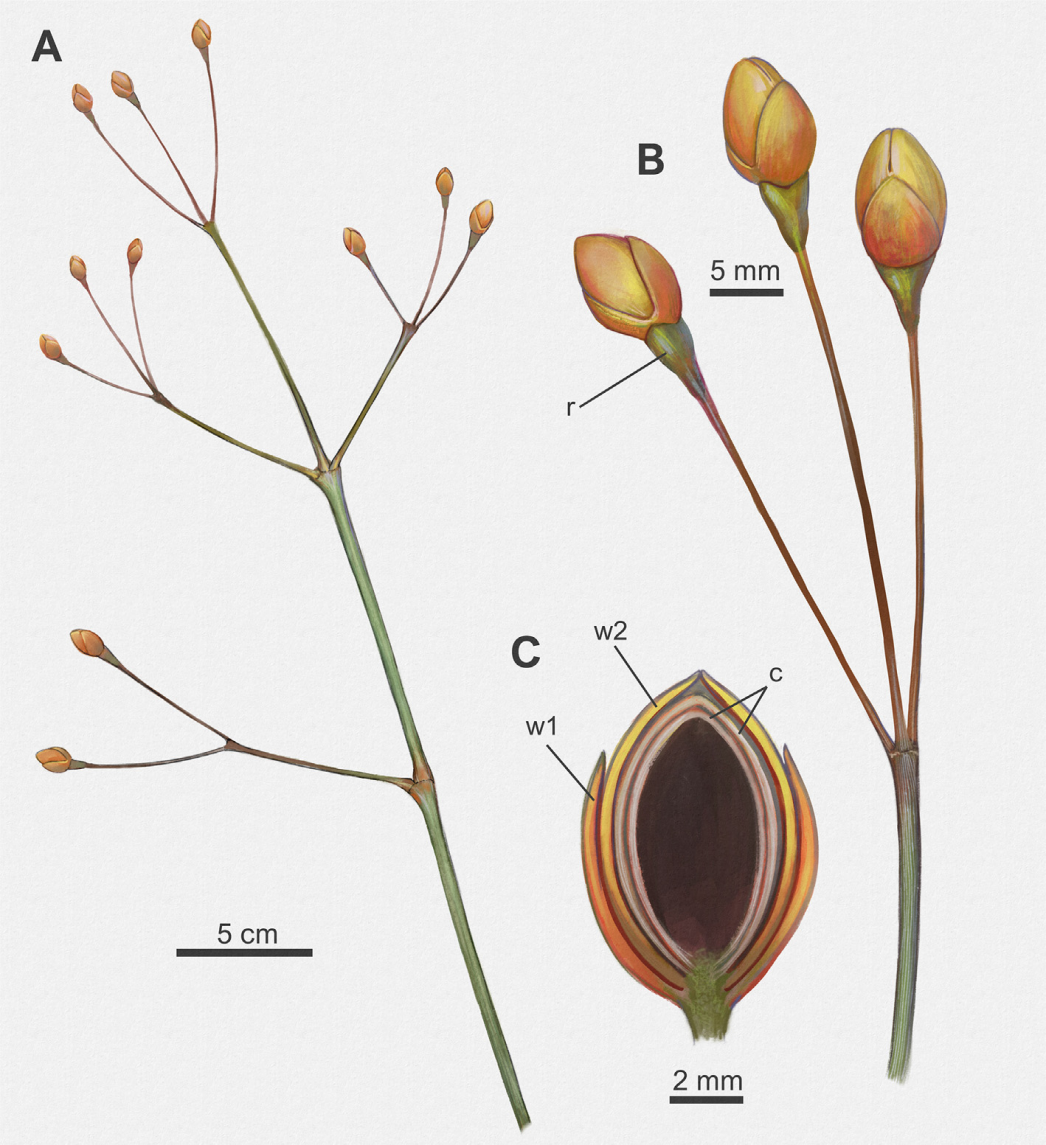
Reconstruction of *Arlenea delicata*. A: Plant portion displaying the habit, opposite branches phyllotaxis, and the terminal female cones; B: Enlarged reproductive branch showing terminal FRUs and detail of swollen receptacle; C: Detail of the FRU showing the pairs of proximal and distal bracts and two ovals internal chlamydosperms. Scale bar = 2 mm (Abbreviations: r, receptacle; w1, proximal whorl of bracts; w2, distal whorl of bracts; c, chlamydosperms). By Júlia d'Oliveira.

The new taxa *Arlenea* *delicata* is a dioecious plant with vegetative and reproductive characters that place it in the Ephedraceae family (Fig. 7). Specifically, *A. delicata* possesses branches with swollen nodes, and longitudinally striated, internodes, with 2–3 opposite branching, reproductive structures terminal to branches, and ovoid to oblong-oval female reproductive units surrounded by 1–2 pairs of bracts with two internal chlamydosperms. It is a small plant like a shrub or sub-shrub. *A. delicata* did not preserve leaves and, as it has copiously branched and delicate branches, the leaves would probably, if present, be scale-like, tiny and without photosynthetic function, as in current *Ephedra* sp., with photosynthesis being a function of the branches (Price, 1996; Santos and Chow, 2014b). *A. delicata* has an affinity to ephedroid fossils from around the world. For example, the furcate and trifurcate branches at the nodes, thin longitudinally arranged striations, and terminal reproductive structures of our new ephedroid specimens are similar to characters described for *Ephedra archaeorhytidosperma* Yang et al. (2005) from the Lower Cretaceous of Northeast China.

The morphology of *Ephedra* sp. may vary between species and sometimes within specimens. Seeds seen in longitudinal section may be lanceolate, elliptical, ovate, or oblong and in cross section they are rounded or angled, and angles are sometimes prominent (Ickert-Bond and Rydin, 2011). In single-seeded cones, the adaxial side of the seed is convex at half the length of the ovule/seed, in two-seeded cones it is usually flat, and in three-seeded cones it has a median longitudinal crest (Ickert-Bond and Rydin, 2011). *Arlenea* *delicata* bears a single seed that is convex in the adaxial region. This is surrounded by two chlamydosperms and has an oblong-oval to ovoid shape. It has an intern median longitudinal crest that goes from the base to the apex of the seed. However, it has a single seed, not three, as the examples cited in Ickert-Bond and Rydin (2011) for the presence of the longitudinal crest. The seed surface of *A. delicata* is smooth, with fine longitudinal grooves, without transverse and undulating protrusions.

*Arleneadelicata* is distinct from other fossil gnetophytes found in the Crato Formation. The ephedroid plants of the Crato Formation, such as *E. paleoamericana*, have reproductive structures bearing numerous whorled bracts (Kerkhoff and Dutra, 2007). In contrast, *A. delicata* has 1–2 pairs of bracts around the female reproductive units. In addition, the branches of *A. delicata* differ from those of *Itajuba* *yansanae* (Ricardi-Branco et al., 2013). *I. yansanae* have 3–4, oppositely decussate branches and have abundant tracheids, exceeding the number of vessels seen in cross-section. *A. delicata*, in contrast, has opposite ramifications of the branches, abundant tracheids with haloed punctuations and vessels with a perforation plate. Furthermore, *A. delicata* possesses oblong-oval female reproductive units, whereas *I. yansanae* carries small and pointed reproductive structures similar to their bracts. *A. delicata* also differs from *F. gracilifolia* in several ways. *F. gracilifolia* has terminal reproductive organs with structures similar to long leaves at the base of the reproductive structures; in addition, *F. gracilifolia* has branches with leaves in organic connection (Löwe et al., 2013) and both male and female reproductive organs. In contrast, *A. delicata* does not have leaves and its reproductive structures are exclusively female.

*Arleneadelicata* is also easily distinguished from ephedroid fossils distributed in Asia. It differs from *Ephedra multinervia* in lacking leaves and axillary female cones (vs. presence of multinerved leaves and normally axillary female cones in *E. multinervia*) (Yang et al., 2015). The seeds of *A. delicata* differ from those of *E. archaeorhytidosperma* in that the latter species has ovules/seeds ornamented with small undulating transverse protuberances (Yang et al., 2005). *Siphonospermum simplex* Rydin and Friis (2010) does not have reproductive units typical of known *Ephedra* plants, such as ovulated cones (Rydin and Friis, 2010). In contrast to *A. delicata*, the chlamydosperms of *S. simplex* are pediceled and lack bracts/subtended leaves. Although *Jianchangia verticillata* Yang et al. (2020) and *A. delicata* have ovulated cones and an inner pair of chlamydosperms, these two fossil plants differ in that *A. delicata* has only two pairs of united bracts that completely surround the chlamydosperms and not multiple pairs of spiral bracts as *J. verticillata* (Yang et al., 2020).

**Fig. 8:**
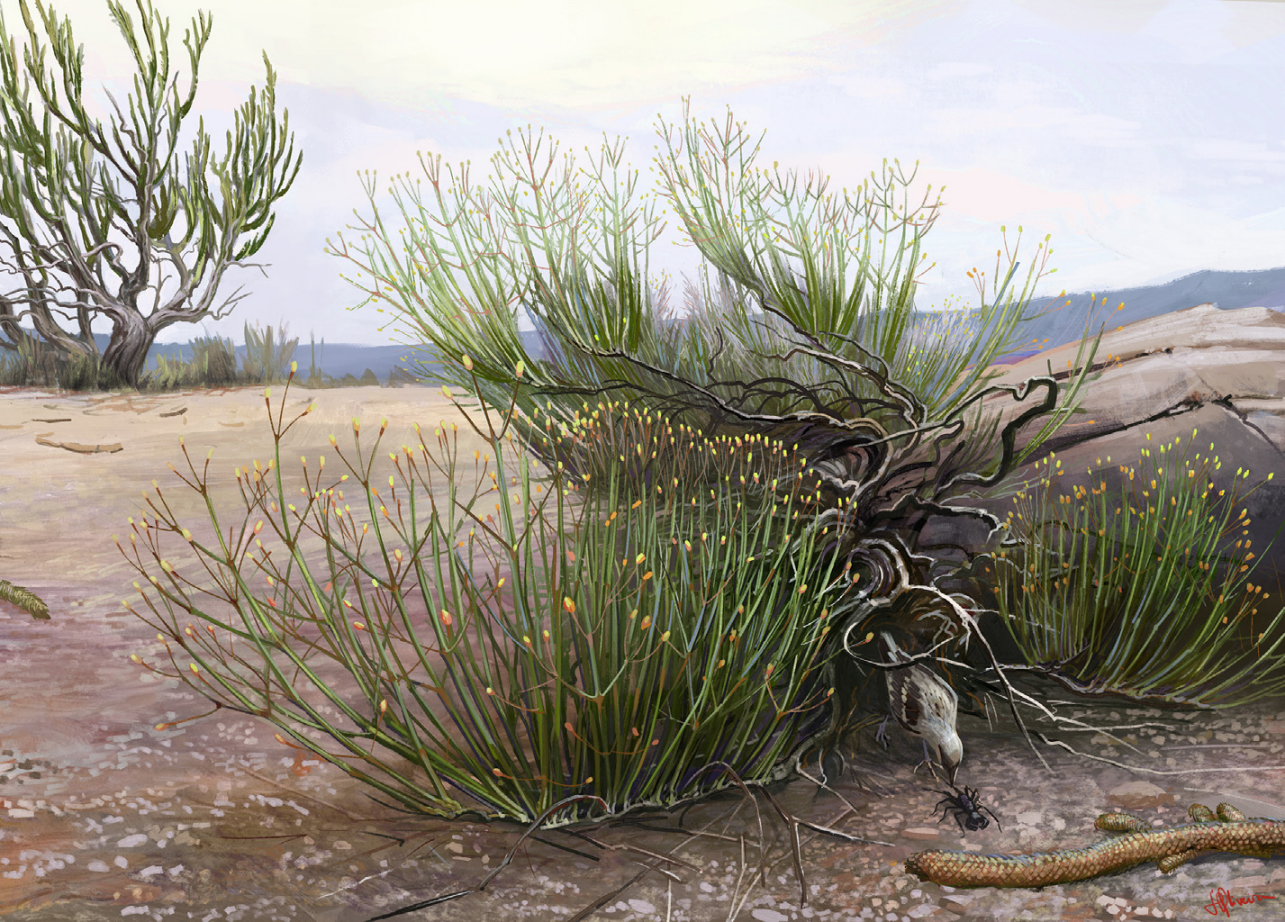
Life reconstruction of *Arlenea delicata* gen. et sp. nov. Diagram by Júlia d'Oliveira.

The morphological characters found in *Arlenea* *delicata* allowed us to infer the climate conditions in which Ephedrales plants flourished in the Cretaceous. Extant *Ephedra* plants occur mainly in deserts, semi-deserts or desert steppes and are generally used as indicators of arid environments (Huang and Price, 2003; Deng, 2007). The morphology and macroscopic anatomy of *A. delicata* suggest that it developed in places that have a water deficit (Fig. 8). The absence of leaves and the abundance of tracheids, similar to those observed in *Itajuba* *yansanae* (Ricardi-Branco et al., 2013), can be interpreted as characteristic adaptations that reduce the evaporative surface of the plant in hot and arid climates. This hypothesis is corroborated by paleoclimatic studies carried out in the Araripe Basin (Heimhofer and Hochuli, 2010), which have indicated that rainfall was sporadic. Some studies have suggested that the first members of the ephedroids may have occupied more diverse habitats, probably also in humid conditions (Sun et al., 2001; Yang et al., 2005, 2020; Fanton et al., 2006; Wang and Zheng, 2010; Yang, 2010).

This new ephedroid fossil also adds evidence that the general morphology of ephedroid plants has remained unchanged since the Cretaceous, even though they have experienced a near-extinction at K/T boundary and twice radiated in the Early Cretaceous and Miocene. This morphological stasis is not unique to Ephedrales, but has also been noted in other plant groups, e.g., *Sassafras* sp., *Liriodendron* sp. and others.

### 4.1 Biogeography and plant dispersion

The consumption of flesh or pulp-covered seeds and subsequent dispersal of those seeds by animals has been an important feature of the reproductive biology of seed plants since Carboniferous (Tiffney, 1984, 1986; Fleming and Lips, 1991). This mutualistic plant–animal interaction is probably the product of diffuse coevolution (Janzen, 1980) in which groups of plants have evolved with groups of animals (Fleming and Williams, 1990). Due to the flourishing forest in the Early Cretaceous around the globe, many vertebrates, including birds, dinosaurs, pterosaurs and mammals, had become arboreal and/or herbivorous (Wang et al., 2005). Among the flying vertebrates, birds and pterosaurs were probably the major competitors for niches and food (Wang et al., 2005).

Extant *Ephedra* sp. have three types of known dispersal agents, which are strongly linked to bract type. Dry-winged membranous bracts are wind-dispersed, leathery bracts are dispersed by seed-collecting rodents, and the fleshy bracts are dispersed by frugivorous birds (Hollander and Vander Wall, 2009; Hollander et al., 2010). *A. delicata* has an enlarged receptacle and fleshy bracts, indicating that its seeds were likely dispersed by animals.

Previous studies have suggested that the fleshy bracts observed in fossil *Ephedra* specimens (e.g., *Ephedra carnosa*) may indicate seed dispersal by early bird species (Yang and Wang, 2013). During the Cretaceous, seeds were dispersed by numerous animals, including both birds and pterosaurs (Fleming and Lips, 1991). Studies of the Yixian Formation in China indicate that the seeds of *Ephedra* sp. may have been dispersed by flying animals, as putative arboreal seed-eaters such as *Sinopterus* sp. and the long-tailed bird *Jeholornis* and some enantiornithes birds occupy similar niches as fossil ephedroids (Wang and Zhou, 2006). Similar to the Yixian Formation, in the fossiliferous layers of the Crato Formation, pterosaur fauna (mainly the Tapejaridae) and some forms of plants (mainly gnetales and angiosperms) occupy the same niches (Early Cretaceous/Aptian) (Mohr et al., 2007; Wang et al., 2012; Lima et al., 2014). The pterosaurs of the Yixian Formation (*Sinopterus* sp.) and those of the Crato Formation (*Tupandactylus* sp.) are morphologically similar, as are the birds *Yixianornis* sp. and *Kaririavis* sp., the latter of which is a possible candidate for plant dispersal in the Aptian of the Crato Formation. Overall, pterosaurs resembled birds in their metabolism, sensory physiology, and flight capabilities (Fleming and Lips, 1991); pterosaur adaptive radiation was ecologically more extensive than indicated by the known fossil record (Fleming and Lips, 1991); and frugivory evolved in the other two groups of flying vertebrates (birds and bats) and is likely to have evolved in pterosaurs with sufficient evolutionary opportunity (Fleming and Lips, 1991). If fleshy bracts and enlarged receptacles do indicate the mode seed dispersal in Ephedrales, this may explain the cosmopolitan distribution of Ephedraceae in the Lower Cretaceous.

## 5 Conclusion

A new ephedroid plant fossil from the Crato Formation, Lower Cretaceous of the Araripe Basin, Brazil is described based on vegetative and reproductive structures. This fossil ephedroid has longitudinally striated branches and branching, reproductive structures terminal to the branches that carry two internal ovoid chlamydosperms subtended by two pairs of bracts. Based on a detailed comparison with fossil and extant ephedroid plants, we assign the present fossils to a new genus and species, *Arlenea* *delicata* gen. et sp. nov., and place it within the family Ephedraceae. The occurrence of *A. delicata* indicates that ephedroids had a wide distribution in Brazil during the Early Cretaceous. The general morphology (e.g., fleshy bracts and receptacles) of this new ephedroid fossil indicate that seeds of this plant were dispersed by animals. This method of seed dispersal would explain the cosmopolitan distribution of Ephedraceae in the Lower Cretaceous.

## Author contributions

AMNR and FJL conceived the project. AMNR and RAMB photographed the fossils specimens. AMNR, YY, AAFS, JTCJ, and FJL analyzed and interpreted the results. RAMB formatted the figures. All authors contributed on drafts and approved the final manuscript.

## Declaration of competing interest

There is no conflict of interest.
